# Flow Cytometry for B-Cell Non-Hodgkin and Hodgkin Lymphomas

**DOI:** 10.3390/cancers17050814

**Published:** 2025-02-26

**Authors:** David C. Gajzer, Jonathan R. Fromm

**Affiliations:** Department of Laboratory Medicine and Pathology, University of Washington, 825 Eastlake Ave E, Seattle, WA 98109, USA; dgajzer@uw.edu

**Keywords:** B-cells, B-cell lymphoma, clonality, flow cytometry, Hodgkin lymphoma, light chain restriction, classic Hodgkin lymphoma, nodular lymphocyte predominant Hodgkin lymphoma, T-cell/histiocyte-rich large B-cell lymphoma

## Abstract

Multi-parametric flow cytometry is a powerful diagnostic tool that permits rapid assessment of cellular antigen expression to quickly provide immunophenotypic information suitable for disease classification. This chapter describes the classification of B-cell non-Hodgkin lymphoma (B-NHL) by flow cytometry suitable for the clinical and research environment. In addition to describing the immunophenotypic patterns of the most common B-NHL (including examples of common B-NHL), the effect of anti-CD19, -CD20, and -CD38 therapies on the evaluation of flow cytometric data is also discussed. Over the last 15 years, our laboratory has developed flow cytometry combinations that can immunophenotype classic Hodgkin lymphoma (CHL), nodular lymphocyte predominant Hodgkin lymphoma (NLPHL), and T-cell/histocyte-rich large B-cell lymphoma (THRLBCL) and the use of these assays will be presented. The CHL assay combination is also particularly well suited to immunophenotype primary mediastinal large B-cell lymphoma (PMLBCL) and our experience immunophenotyping PMLBCL by flow cytometry will be discussed. Finally, an approach to the evaluation of the reactive infiltrate of CHL, NLPHL, and THRLBCL that can provide diagnostic information will also be provided.

## Simple Summary

Flow cytometry is a cell-based technique that helps provide diagnostic information in the hematopathology laboratory. In this review, we discussed how the technique can be used to classify both B-cell lymphomas and Hodgkin lymphoma. For B-cell lymphomas, emphasis is placed on the use of B-cell markers (CD19 and CD20) and other critical antigens (CD5, CD10, and CD38) for classification. Furthermore, the role of flow cytometry in measurable residual disease detection and evaluation of specimens after directed therapies is discussed. The approach to immunophenotyping the neoplastic cells of classic Hodgkin lymphoma and related lymphomas is also presented. The non-neoplastic cells that predominate in Hodgkin lymphoma and related lymphomas tend to show unique antigen expression profiles when compared to the expression profiles of non-neoplastic tissues. The use of these differences in expression profiles for diagnostic purposes will also be discussed.

## 1. Introduction

Mature B-cell neoplasms form a group of disorders with highly variable clinical presentations, pathologic features and prognoses, but also some common features. Flow cytometry (FC) allows for rapid and sensitive assessment of the immunophenotype of individual cells in a mixed suspension, thereby allowing for identification of abnormal cell populations, even amongst an abundant reactive infiltrate [1]. The technique is used to evaluate hematopoietic neoplasms from a variety of liquid specimen types (disaggregated tissues, bone marrow, peripheral blood, body fluids). In this review, we will discuss the role of flow cytometry in the diagnosis and subclassification of mature B-cell non-Hodgkin lymphoma and Hodgkin lymphoma, evaluation of disease prognosis, and evaluation of minimal/measure residual disease after treatment. The manuscript focuses on the current approach B-NHL evaluation at the University of Washington; readers are directed to other reviews for approaches by other authors [2,3,4]. Additionally, some specific uncommon entities are not covered herein and readers are advised to consult more comprehensive compilations [5,6]. Finally, a discussion of the specific FC combinations used, methods for processing, and data analysis can be found elsewhere [7,8].

## 2. Diagnosis of Mature B-Cell Neoplasms by Flow Cytometry

**Figure 1:**
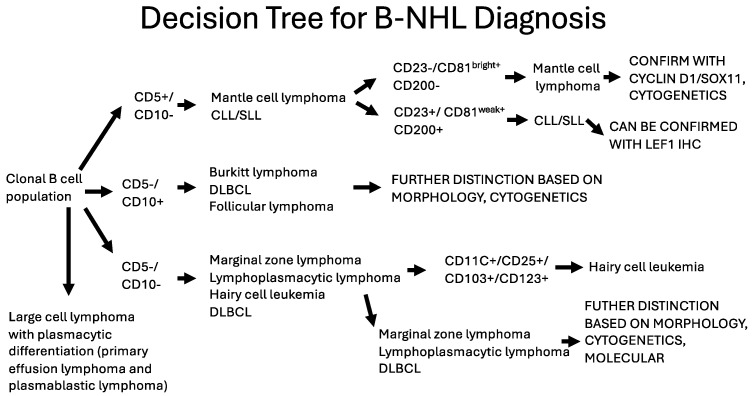
Schematic showing an approach to the evaluation of B-cell NHL workup by FC. Evaluation of B-NHL can be divided into those that express CD5 without CD10, those that express CD10 without CD5, those that are negative for CD5 and CD10 and those comprised of large cells with plasmacytic differentiation. Exceptions to the schematic exist and details of the evaluation are provided in the text.

The initial evaluation of the B-cells begins with establishing expression of a lineage defining B-cell marker such as CD19 or CD79b on populations of interest. Kappa and lambda light chains are typically included in the analysis and allow for the identification of clonal or reactive populations as the normal, reactive kappa to lambda ratio is approximately 1.5 to 1. In neoplastic populations, the ratio is often significantly higher or lower, depending on whether the neoplastic population expresses kappa or lambda light chains. More specificity is typically obtained with the inclusion of additional antigens (for example, CD5, CD10, and CD38; see below) that allows for the identification of populations with immunophenotypes that differ significantly from normal. Subsequent sections will describe in greater detail the utility of antigens for classification. See Figure 1 for a summary of the approach to B-cell NHL disease classification and Table 1 which summarizes the immunophenotypes for the most common B-cell lymphomas (also described in detail below).

## 3. Defining B-Cell Lineage

Antigens useful for defining B-cell lineage include (but are not limited to) CD19, CD20, CD22, and CD79a and CD79b. Of these, CD19 has proven to be the most useful marker because it is both lineage specific and expressed during all phases of B-cell maturation. Moreover, CD19 is expressed by most mature B-cell lymphomas; important exceptions include plasma cell myeloma (where CD19 expression is aberrantly decreased) and other lymphoplasmacytoid lymphomas of B-cell origin (for example, plasmablastic lymphoma and primary effusion lymphoma; see below). CD79a (present in the cytoplasm) and CD79b (present on the surface) are also expressed throughout B-cell maturation, although some variability of expression on/in B-cell lymphomas may be observed. Expression of CD20 is initially seen on late-stage B lymphoblasts with robust expression detected on mature B-cells; expression of CD20 decreases as cells differentiate to plasma cells. Given that CD20 is expressed on more mature B-cells, evaluation of the antigen with other markers (such as CD10 and CD38) define normal patterns of B-cell maturation, particularly important for evaluation of B-cell lymphoma in the bone marrow by flow cytometry, where normal B lymphoblasts could be mistaken for B-cell lymphoma. See the listed reference ([1]) for a more comprehensive description of B-cell maturation. It is important to note that CD20 is characteristically decreased or absent in chronic lymphocytic leukemia/small lymphocytic lymphoma (CLL/SLL); likewise, some large B-cell lymphoma will lack expression of CD20 [9] and even T-cell lymphomas can (rarely) express the antigen [10]. CD22 is often used by practitioners of flow cytometry to define B-cells; however, CLL/SLL, follicular lymphoma and some large B-cell lymphomas can show low or very low expression of the antigen, and other B-cell lymphomas (such as hairy cell leukemia) show bright expression of CD22 [11].

Finally, it should be noted that the B-cell lymphomas discussed in this manuscript are mature and so (with only rare exceptions) do not express markers of immaturity (CD34 and TdT). A discussion of immature B-cell neoplasms (B lymphoblastic leukemia/lymphoma) is beyond the scope of this review. In addition, readers interested in practical caveats associated with B-cell lymphoma analysis by flow cytometry are directed to reviews on this topic [1,12].

## 4. CD5-Positive/CD10-Negative Small B-Cell Neoplasms

**Figure 2:**
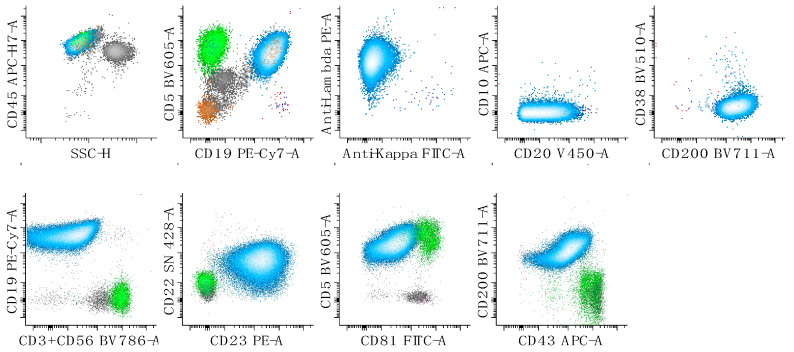
Flow cytometric characterization of a case of chronic lymphocytic leukemia/small lymphocytic lymphoma (CLL/SLL). The neoplastic population (cyan) demonstrates expression of lambda surface light chains, CD5, CD19, CD20 (decreased), CD22 (low), CD23, CD43, CD45, CD81 (low), and CD200 without expression of CD10, CD38, or CD3/CD56. The first dot plot of the top panel shows all cells, the second shows lymphocytes, and the last three show only B-cells. The bottom panels of dot plots show lymphocytes. Reactive T-cells are colored in green.

**Figure 3:**
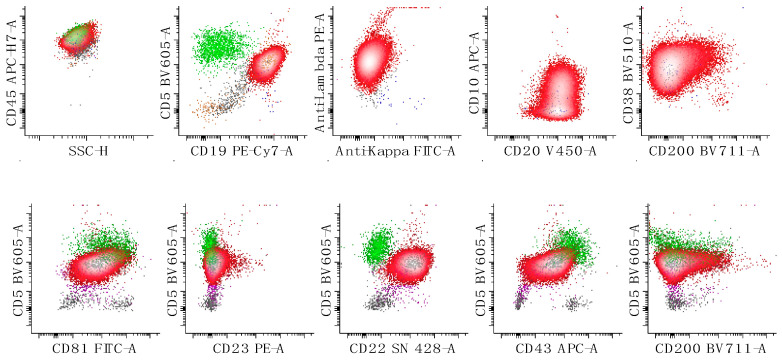
Flow cytometric characterization of a case of mantle cell lymphoma. The neoplastic population is colored in red and demonstrates expression of lambda surface light chains, CD5, CD19, CD20, CD22, CD38, CD43, CD45, CD81, CD200 (low to negative) and no expression of CD23. CD10 may be expressed at a very low level. The first dot plot of the top panel shows all cells, the second shows lymphocytes, and the last three show only B-cells. The bottom panels of dot plots show lymphocytes. Reactive T-cells are colored in green. The small kappa expressing B-cell population is in blue and lambda-expressing B-cells are in red. All other events are in grey.

The differential diagnoses among CD5-positive B-cell neoplasms include mainly chronic lymphocytic leukemia/small lymphocytic lymphoma (CLL/SLL) (example, Figure 2), mantle cell lymphoma (MCL) (example, Figure 3) and (rarely) marginal zone lymphoma (MZL) [13]. Characteristically, CLL/SLL features expression of variably decreased CD20, CD5, decreased CD81, CD23, CD43, bright CD200, dim to absent FMC7, and mono- or bi-clonal, dim or negative surface immunoglobulin expression [14].

MCL typically expresses bright CD20 and has expression of CD5 and CD81, while CD200 is dim to absent; surface immunoglobulin expression tends to be brighter than in CLL/SLL and often demonstrates lambda light chain restriction. Of note, a small minority of cases of MCL express CD200, are associated with hypermutated IGVH genes and a lack of SOX-11 expression by immunohistochemistry and are characterized by an indolent clinical course [3]. If a CD5+ B-cell lymphoma demonstrates an immunophenotype atypical for CLL/SLL and MCL, a CD5-positive MZL should be considered [15]. While our laboratory has observed that MZL is typically characterized by relatively bright expression of CD32 and CD44 (unpublished data), correlation with histopathology and immunohistochemistry is mandatory for a definitive diagnosis. Tissue section morphology and immunohistochemistry (IHC) are also critical for confirming a diagnosis of MCL (cyclin D1, SOX11-positive by IHC; presence of t(11;14) by fluorescence in situ hybridization) and CLL (LEF1 by IHC). Finally, FC has a role in disease prognostication in CLL; CD49d expression status by FC has been reported to be one of the most important independent prognostic factors in patients with CLL/SLL in addition to TP53 and IGVH mutation status [16].

The detection of low-level monotypic B-cell populations requires clinical and laboratory correlation, with those cases characterized by <5 × 10^−3^ abnormal cells/μL in the peripheral blood and no overt clinical evidence of a hematolymphoid neoplasm being compatible with a diagnosis of monoclonal B-cell lymphocytosis (MBL). Abnormal B-cell populations without a CLL/SLL-like or hairy cell leukemia (HCL)-like immunophenotype can qualify as a non-CLL type MBL in the absence of splenomegaly [17]. Similarly, small, abnormal B-cell populations identified in normal or minimally enlarged lymph nodes are regarded as being the tissue equivalent to peripheral blood MBL [18].

## 5. CD5-Negative/CD10-Negative Small B-Cell Neoplasms

**Figure 4:**
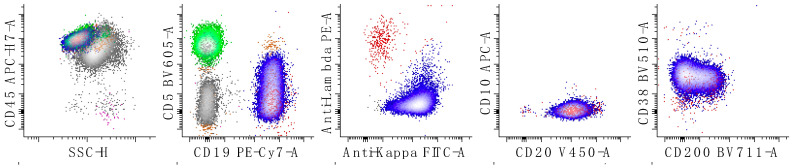
Characterization of a case of marginal zone lymphoma by flow cytometry. The neoplastic population (blue due to expression of kappa light chains) shows expression of CD19, CD20, and CD45 without expression of CD10 or lambda light chains. CD5 and CD200 are likely expressed at low levels. T-cells are green and kappa and lambda-expressing B-cells are in blue and red, respectively. The first dot plot shows all cells, the second all lymphocytes, and the third through fifth show only B-cells.

**Figure 5:**
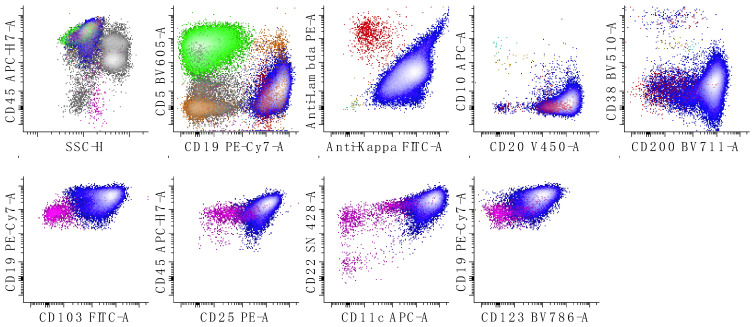
Immunophenotypic characterization of a case of hairy cell leukemia in the bone marrow. The neoplastic hairy cell leukemia population (blue in top panel due to kappa light chain expression) demonstrates increased side light scatter (compared to the T-cells) and expression of CD11c, CD19, CD20 (bright), CD25, CD38 (low), CD45 (increased), CD103, CD123, and CD200 (bright) without CD5 or CD10. In the top row of dot plots, the first dot plot shows all leukocytes, the second all lymphocytes, and the remaining three dot plots show only B-cells. The bottom rows of plots show only B-cells. Top panel of plots: T-cells are colored in green, kappa-restricted B-cells are colored in blue, and the lambda-restricted B-cells are colored in red. Bottom panel: neoplastic population in blue, and remaining B-cells in magenta. All other events are in grey. A small population of co-incident events is shown in orange.

Assessment of CD5-negative/CD10-negative B-cell NHL poses a diagnostic challenge, with the main diagnostic considerations being marginal zone lymphoma (MZL (Figure 4)) including splenic marginal zone lymphoma [SMZL], which represents a subset of MZL), splenic diffuse red pulp small B-cell lymphoma, CD10-negative follicular lymphoma, lymphoplasmacytic lymphoma (LPL), hairy cell leukemia (HCL) (Figure 5) and hairy cell leukemia variant (HCLv) (International Consensus Classification)/splenic B-cell lymphoma/leukemia with prominent nucleoli (WHO, 5th edition). Additionally, CD5-negative CLL/SLL would also be a consideration in this category.

In addition to expression of B-cell markers, SMZL can show expression of CD11c typically without CD103 or CD123 expression [19]. “Typical” MZL (nodal and extranodal) also shows expression of B-cell antigens, the absence of CD5 and CD10 (in most cases), and expression of CD43 in a minority of cases; MZL historically has shown significant immunophenotypic overlap with LPL (discussed below). Work by Mason and colleagues from 2017 provides some guidance to distinguish MZL from LPL [20]. MZL expresses CD1d but is largely negative for CD200; conversely, LPL is low to negative for CD1d but expresses CD200. LPL also typically lacks expression of CD103 and CD123 and shows lymphoplasmacytoid cytomorphology. Importantly, the *MYD88* L265P mutation has been demonstrated in the vast majority of LPL cases (90%). While it is not completely specific, examination for the *MYD88* L265P mutation is routine in clinical practice because the immunophenotype of LPL can overlap other B-cell lymphomas that lack expression of CD5 and CD10 [21]. HCL is characterized by expression of CD11c, CD22, CD25, CD103 and CD123 by FC [22]. In our experience, this immunophenotype is quite specific for HCL, although occasional cases will show expression of CD10. While the *BRAF* V600E mutation is characteristic (but not diagnostic) of HCL, the specificity of the flow cytometric immunophenotype often makes molecular testing in HCL unnecessary [23]. HCL-v/splenic B-cell lymphoma/leukemia with prominent nucleoli demonstrates expression of CD11c and CD103 (but in contrast to HCL) lacks expression of CD25 and CD123 [22].

FC can be useful in helping to distinguish B-cell lymphoma with extreme plasmacytic differentiation (like MZL or LPL) from a true plasma cell neoplasm. First, a B-cell lymphoma with plasmacytic differentiation will often show both distinct abnormal B-cell and plasma cell components, while a PCN will only show an abnormal plasma cell population. Additionally, expression of CD19 and CD45 are often aberrantly lost from PCN but expressed by MZL or LPL. Finally, the plasma cell component of B-cell lymphoma with plasmacytic differentiation usually lacks expression of CD56 (in contrast to PCN) [24]. Readers interested in morphologic features that support the different types of CD5-/CD10- B-NHL are referred to relevant reviews [25].

## 6. CD10+/CD5- B-Cell Lymphomas

The main CD10-positive/CD5-negative entities that can be characterized by FC include follicular lymphoma, Burkitt lymphoma, and a subset of diffuse large B-cell lymphoma, NOS and high-grade B-cell lymphomas. The latter two entities are discussed in a separate section below.

**Figure 6:**
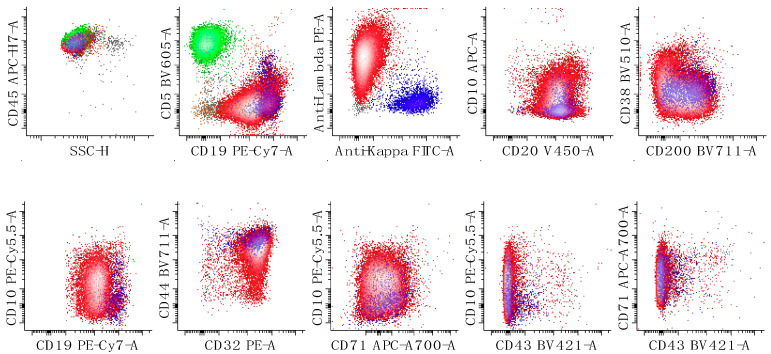
Immunophenotypic characterization of a case of follicular lymphoma (grade 1; classic follicular lymphoma). The neoplastic cells show expression of lambda surface light chains, CD10, CD19 (decreased expression relative to the reactive B-cells), CD20, CD32, CD38 (decreased relative to the expression of a normal germinal center population [29]), CD44 (decreased), CD45 and CD71 (at the level of the normal B-cells) with no expression of CD5 or CD43. The first dot plots of the top row show all leukocytes, the second shows all lymphocytes, and the last three show only B-cells. The bottom rows of dot plots show all B-cells. T-cells are colored in green, kappa-restricted B-cells are colored in blue, and lambda-restricted B-cells are colored in red. Neoplastic B-cells are red due to lambda light chain expression. A small number of granulocytes is shown in grey. A discussion of using CD32, CD44, and CD71 to immunophenotype B-cell lymphoma is presented elsewhere [33].

Follicular lymphomas (with grades 1–2 and grade 3A, now called classic follicular lymphoma and grade 3B now called follicular large B-cell lymphoma in the WHO classification system, respectively) demonstrate expression of CD10, (in approximately 90% of cases [26]), B-cell antigens (including CD19 and CD20), CD38, surface light chain restriction typically without expression of CD5 (Table 1). Of note, decreased expression of CD19 [27,28] and lower expression of CD38 (when compared to the expression in reactive germinal centers) has been observed [29]. The surface expression of light chains can often be low [30]. Finally, most cases demonstrate expression of BCL2 [31], although most cases can be confidently immunophenotyped without evaluating expression of this antigen. We and others have demonstrated normal levels of expression of CD71 (relative to normal resting lymphocytes) in contrast to diffuse large B-cell lymphoma [32,33,34]. While FC is useful for immunophenotyping this lymphoma, correlation of these findings with tissue section morphology is required for a definitive diagnosis of follicular lymphoma. An example of a case of follicular lymphoma is shown in Figure 6.

**Figure 7:**
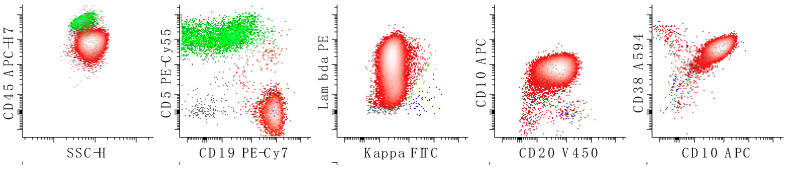
Immunophenotypic characterization of a case Burkitt lymphoma. The neoplastic cells show expression lambda surface light chain restriction, CD10, CD19, CD20, CD38 (increased; compare to expression seen with follicular lymphoma, Figure 6), and CD45 (decreased relative to T-cells) with no expression of CD5. The first dot plot shows all leukocytes, the second shows all lymphocytes, and the last three show only B-cells. T-cells are colored in green, kappa-restricted B-cells are colored in blue, and lambda-restricted B-cells are colored in red. Neoplastic B-cells are red due to lambda light chain expression. All other remaining events are in grey.

Burkitt lymphoma typically also expresses CD10 and B-cell markers, surface light chain restriction, relatively bright expression of CD38, and CD43, without expression of CD5. BCL-2 expression is typically absent. The diagnosis must be confirmed with tissue section morphology and immunohistochemistry. Evidence of the MYC translocation should also be confirmed by fluorescence in situ hybridization (FISH) [35]. An example of a case of Burkitt lymphoma is shown in Figure 7.

## 7. Large B-Cell Neoplasms/High-Grade B-Cell Lymphoma

**Figure 8:**
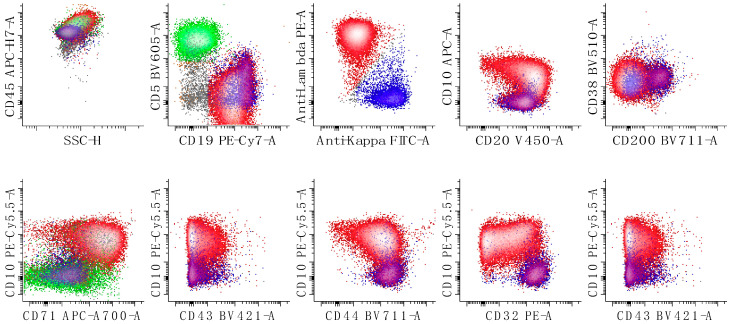
Immunophenotypic characterization of a case of diffuse large B-cell lymphoma. The neoplastic cells show expression of lambda surface light chain restriction, CD10, CD19 (decreased expression relatively to the reactive B-cells), CD20, CD32 (decreased), CD38 (low), CD44 (decreased), CD45 (increased) and CD71 (increased relative to normal B-cells) with no expression of CD5 or CD43. CD71 expression is also increased relative to that seen in follicular lymphoma (Figure 6). The first dot plots of the top row show all leukocytes, the second shows all lymphocytes, and the last three show only B-cells. The bottom rows of dot plots show all B-cells. T-cells are colored in green, kappa-restricted B-cells are colored in blue, and lambda-restricted B-cells are colored in red. Neoplastic B-cells are red due to lambda light chain expression. All other events are in grey.

While FC is not the foremost diagnostic test for characterization of most large B-cell lymphomas (as the immunophenotypes can vary and the diagnosis requires identification of a characteristic morphology), it can often identify certain distinctive immunophenotypic and light scatter features (see Table 1). One well-known limiting factor in the evaluation of large B-cell neoplasms is a spuriously negative FC result in a sample prepared from material containing frank neoplasm, possibly due to increased cell turnover or a scarcity of large, neoplastic B-cells (like in entities such as T-cell/histiocyte-rich large B-cell lymphoma, see below) [36]. Rapid and gentle tissue processing may help alleviate some of these limitations. The sizes of the neoplastic B-cells can generally be estimated by their forward light scatter properties, and this is especially useful for disaggregated cytology specimens. Additionally, as was noted above, diffuse large B-cell lymphomas tend to have greater expression of CD71 than low-grade lymphomas (see example with increased CD71, Figure 8) [32,33,34]. FC may also prove useful in distinguishing a mature B-cell neoplasm from B-lymphoblastic leukemia/lymphoma (B-LL). Expression of immaturity-associated antigens such as CD34 and TdT as well as decreased expression of CD20 and CD45 are helpful in this regard (arguing for B-LL and against a mature B-cell lymphoma). One caveat in this regard is the fact that high-grade B-cell lymphomas (usually “double hit” lymphomas) tend to demonstrate a lower expression level of CD20 and CD45 [37] and may rarely express TdT. Such TdT expressing high-grade B-cell lymphomas typically represent transformed follicular lymphoma [38].

## 8. Large B-Cell Lymphomas with Plasmacytic Differentiation

**Figure 9:**
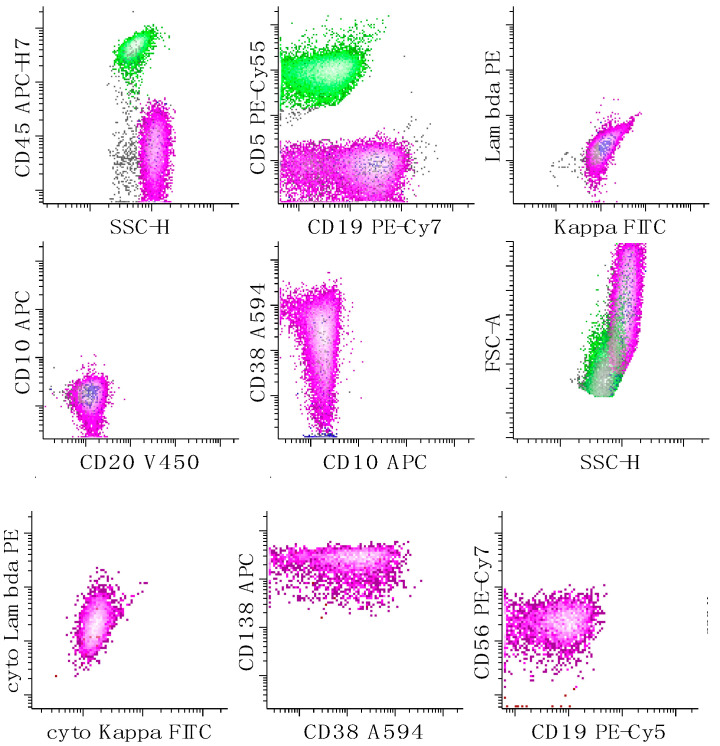
Immunophenotypic characterization of a case of plasmablastic lymphoma by FC. This neoplasm is characterized by both a B-cell tube (top two rows of dot plots) and a plasma cell tube (bottom row of dot plots). The neoplastic population (magenta) shows large cell size (as measured by increased forward light scatter), probable lambda cytoplasmic light chains, CD38 (variable), CD56 (low), and CD138 without expression surface light chains, CD10, CD20 or CD45. The neoplastic population also has expression of CD19 in the B-cell tube; the reason for the apparent lack of expression of CD19 in the plasma cell tube is unclear. T-cells are colored in green. All other events are in grey.

FC can be helpful in immunophenotyping plasmablastic lymphoma and primary effusion lymphoma, although assessment of histologic sections or a cell block preparation and immunohistochemistry studies on these materials is required. Plasmablastic lymphoma (example, Figure 9) and primary effusion lymphoma share immunophenotypic and clinical features, such as an association with immunodeficiency. Plasmablastic lymphomas demonstrate differentiation toward plasma cells and thus often lack CD20 and express bright CD38 and CD138 by FC and demonstrate expression of MUM-1, Ki-67 (proliferation rate approaching 100%) and c-MYC by immunohistochemistry [39]. Primary effusion lymphoma tends to demonstrate expression of activation markers CD30 and CD38, often without pan B-cell marker expression (CD19, CD20, and CD79a). CD45 and CD138 expression is variable; by immunohistochemistry/in situ hybridization, positivity for HHV-8 and EBER are characteristic features [40].

## 9. Reactive Versus Neoplastic Clonal B-Cell Processes

It is important to note that not every clonal B-cell population identified by FC represents B-cell lymphoma. Reactive B-cell clones have been observed in Hashimoto’s thyroiditis [41] and in lymph node biopsies showing follicular hyperplasia [42]. Importantly, reactive clonal B-cell populations tend to have an otherwise normal immunophenotype, in contrast with neoplastic B-cell populations, which show immunophenotypic abnormalities of other antigens (for instance CD5, CD10, CD38, and CD45), resulting in immunophenotypic differences from normal populations. Small abnormal B-cell populations may represent monoclonal B-cell lymphocytosis (see above) [17,18,43] or in situ follicular neoplasia (often now called in situ follicular B-cell neoplasm), a precursor lesion of follicular lymphoma showing colonization of morphologically normal geminal centers that harbor the t(14;18) of follicular lymphoma and aberrantly co-express CD10 and BCL-2 [44,45].

## 10. Evaluation of B-Cell Non-Hodgkin Lymphoma Measurable Residual Disease (MRD) and Consequences of Directed Therapies by Flow Cytometry

**Figure 10:**
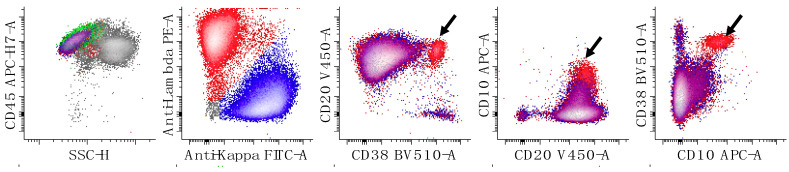
Minimal residual disease detection of high-grade B-cell lymphoma, NOS in the peripheral blood. Overall, this peripheral blood specimen demonstrates a normal kappa to lambda light chain ratio of 1.2. In addition, multiple dot plots do not demonstrate an abnormal B-cell population. However, a small (0.2% of leukocytes) lambda-restricted B-cell population is noted (arrows) with expression of CD10, CD19 CD20, CD38 (increased), and CD45 without CD5 or CD200. Note that the large polyclonal collection of mature B-cells (12.3% of the leukocytes) is represented by a mixture of blue (kappa-expressing) and red (lambda-expressing) events, while the small clonal kappa expressing abnormal B-cell population is present as a collection of red events in multiple projections of the data. The first dot plot shows all leukocytes, while the remaining four dot plots show only B-cells. T-cells are colored green, and the remaining cells are in gray.

While the bulk of this chapter describes the use of FC for the initial diagnostic evaluation of B-cell lymphomas, this technique can also be used to routinely evaluate for measurable residual disease (MRD; example of MRD evaluation of a CD10+ high-grade B-cell lymphoma is shown in Figure 10). Evaluation of MRD has become important as the ever-increasing number of chemotherapies and directed therapies can reduce disease burden; being able to measure neoplastic populations after therapy that is below the limited of morphologic detection is useful and likely will become increasingly important in the future to help determine timely therapeutic interventions [46].

Associations between event-free and overall survival and FC MRD status in CLL/SLL have been demonstrated [47,48]. The approach to evaluating MRD for B-cell lymphoma by FC relies on a “difference from normal” technique that underlies the evaluation of MRD for both leukemias and lymphomas [1]. Briefly, the approach requires FC combinations with sufficient differences in antigen expression between normal and small abnormal populations (for example, expression of CD5 and decreased CD20 in the case of chronic lymphocytic leukemia/small lymphocytic lymphoma). Understandably, the technique requires the practitioner to be well versed in the normal patterns of antigen expression in all specimens types for which technique is performed. The evaluation of B-NHL MRD is typically easier than with other hematopoietic neoplasms (like acute myeloid leukemia), as the evaluation of surface light chains provides an identification of a light chain restricted population with an aberrant immunophenotype. Assuming adequate cells can be acquired, sensitivities of 10^−4^ or better can be achieved [49,50]. Details and protocols encompassing this approach are beyond the scope of this review and interested readers are directed to reviews on this topic [12,51]. Corresponding molecular methods for evaluating B-NHL MRD can be found in various reviews [52].

Directed therapies against cell surface antigens have resulted in unique difficulties in data evaluation for the hematopathologist, as these therapies often resulting in no expression of the targeted antigen. The mechanisms for this are varied and include phagocytosis of antibody-bound antigens and blocking of targeted antigen by the therapeutic antibody [3,53]. Given that many of the targeted antigens that are critical for identification and lineage assignment of B-cell populations and determining whether the population in question is immunophenotypically aberrant, evaluation of these population can be challenging and in some cases, require new strategies to identify abnormal populations. In our practice, anti-CD20, anti-CD19, and (in selected cases) anti-CD38 therapies are used most commonly and have the most profound effect on B-NHL flow cytometric evaluation.

In 1997, rituximab (chimeric mouse/human IgG1 monoclonal antibody directed against the extracellular loop of CD20) was approved for treatment of B-NHL, particularly relevant as most B-cell NHL express CD20 [54]. Consequently, the drug is used to treat many different types of B-NHL. Rituximab mechanism of action is due to antibody-dependent cell cytotoxicity, complement mediated cytotoxicity, and antibody-dependent cell phagocytosis [54,55]. Practitioners of diagnostic FC will note the absence of CD20 on B-cells from patients treated with rituximab can complicate data analysis.

Anti-CD19 directed therapies are analogous to CD20-directed therapies, although differ in some important ways. CD20 is expressed on mature B-cells and mature B-cell neoplasms, while CD19 expression is observed on immature B-cells, mature B-cells, plasma cells, immature B-cell neoplasms (B lymphoblastic leukemia/lymphoma) and mature B-cell lymphomas [1]. Two commonly used anti-CD19 therapies, anti-CD19 CAR T-cells (chimeric antigen receptor T-cells; patient T-cells engineered to react to a specific therapeutic target antigen) and blinatumomab (bispecific T-cell engager antibody that reacts with CD3^+^ T-cells and CD19^+^ B-cells to link the cells and activate cytotoxic activity of the T-cells) can result in decreased or absent expression of CD19 [56,57]. Frequently, anti-CD19 and anti-CD20 directed therapies are used together. As CD19 and CD20 are frequently used to define B-cells by FC, new approaches are needed to identify B-cells. While not as specific as CD19 and CD20, our laboratory uses a novel combination of CD22, CD40, and HLA-DR to define B-cells (unpublished data). A study collecting the data from the use of that combination in clinical practice is currently underway.

**Figure 11:**
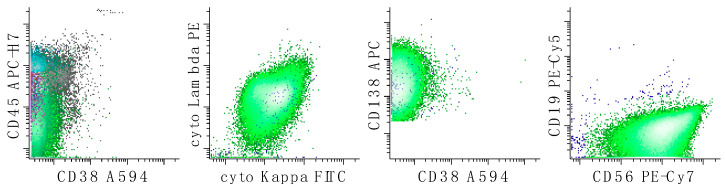
Example of a plasma cell neoplasm treated with anti-CD38 therapy. The plasma cell neoplasm demonstrates expression of CD56, CD138 and ambiguous light chains without expression of CD38 or CD45. As there is very frequent expression of CD38 on de novo plasma cell neoplasms, the lack of CD38 expression is due to prior anti-CD38 therapy. As most gating strategies for plasma cell neoplasms incorporate expression of CD38, identifying plasma cell neoplasms can be more challenging in the setting of anti-CD38 therapy. The first plot shows all events; the last three plots show only plasma cells. Plasma cell neoplasms in green. A very small collection of cellular debris is in blue; all other events are in gray.

Anti-CD38 therapies (Daratumab, Isatuximab; monoclonal antibodies directed again CD38) are principally used to treat plasma cell neoplasms [58,59]. While plasma cell neoplasms (PCN) are not formally covered in this review, we have included a discussion and an example of a PCN treated with Daratumab, as a number of non-PCN neoplasms are now being treated with the drug as part of clinical trials [60] and because of the profound effect of treatment on the flow cytometric data analysis. Patients treated with Daratumab will show cells that lack expression of CD38. In the case of plasma cell neoplasms (example shown in Figure 11), another plasma cell marker is needed to identify the plasma cells (CD138, BCMA, etc.) or a CD38 antibody that is not blocked by Daratumab (for example, clone JK36). Occasionally other B-cell-derived neoplasms (B-cell lymphomas including large B-cell lymphomas with plasma cell differentiation (for example, plasmablastic lymphoma) and B-lymphoblastic leukemia/lymphoma) will be treated with an anti-CD38 therapy. Care must be taken to not regard the lack of CD38 as aberrant.

## 11. Flow Cytometry to Identify the Neoplastic Cells of Classic Hodgkin Lymphoma

Classic Hodgkin lymphoma (CHL) is unique type of B-cell lymphoma [61,62,63,64] in which: (1) the neoplastic Hodgkin and Reed–Sternberg (HRS) cells are rare (less than 1% of the cells in lymph nodes) [64,65], (2) the bulk of the cells in an involved lymph node include reactive lymphocytes, plasma cells, histiocytes and eosinophils [64], and (3) the HRS cells in tissue bind to reactive CD4^+^ T-cells, resulting in HRS cell-T-cell rosettes [66,67,68,69,70,71,72]. CHL has historically been diagnosed by morphology and immunohistochemistry. The immunophenotype of HRS cells differs from other B-cell lymphomas, showing expression of CD15 and CD30 but lacking expression of CD20, CD3, and CD45 [64,73,74]. Studies in our laboratory, however, have demonstrated that HRS cells can be immunophenotyped by FC with high sensitivity and specificity (89 and 100%, respectively), providing useful diagnostic information [72,75,76,77,78] quickly and at a lower cost than immunohistochemistry. Similar results were recently reported by another group (using the antibodies shown in Table 2 adapted to their flow cytometer) providing a CHL FC assay with high sensitivity (95.4%) and specificity (98.2%), respectively [79]. As T-cells bind (rosette) HRS cells and the interaction can be detected by FC, a characteristic feature of these studies is the identification of cells with expression of both T-cell and HRS antigens [72,75,77,78].

**Figure 12:**
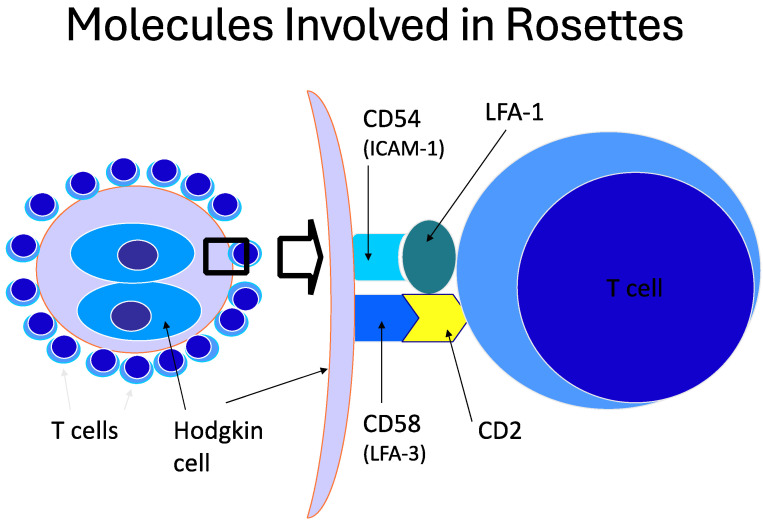
Schematic showing the interaction of Hodgkin cells with T-cells (rosetting). T-cells are bound to the surface of Hodgkin’s cells through the interaction of adhesion molecule pairs (CD 54 -LFA-1, and CD58-CD2) [70,72]. This interaction is responsible for HRS cells appearing to have expression of T-cell antigens by FC. Work in our laboratory also demonstrates that patients with an increased number of T-cell-HRS cell rosettes have a better outcome than CHL cases with lower numbers of these interacting cells [86].

If purified HRS cells are needed for genetic studies, unlabeled “blocking” antibodies (antibodies that can compete for the binding of the adhesion molecule binding partner [70,72]) can be used to abrogate (“block”) T-cell-HRS cell rosetting by disrupting the interaction of CD54 and CD58 on the HRS cells with LFA-1 and CD2, respectively on the T-cell (Figure 12). These studies also provide direct evidence for the role of these adhesion molecules in the HRS cell-T-cell interaction.

We do not recommend using blocking antibodies in routine clinical diagnostic practice, as the observation of the presence of T-cell-HRS cell rosettes is diagnostically useful [75]. A reagent combination for 9-color [75] FC platforms is shown in Table 2. Note further that our laboratory is currently validating a 12-color CHL assay for clinical use. Primary mediastinal large B-cell lymphoma (PMLBCL) can also be immunophenotyped with the 9-color FC combination for CHL (see below).

**Figure 13:**
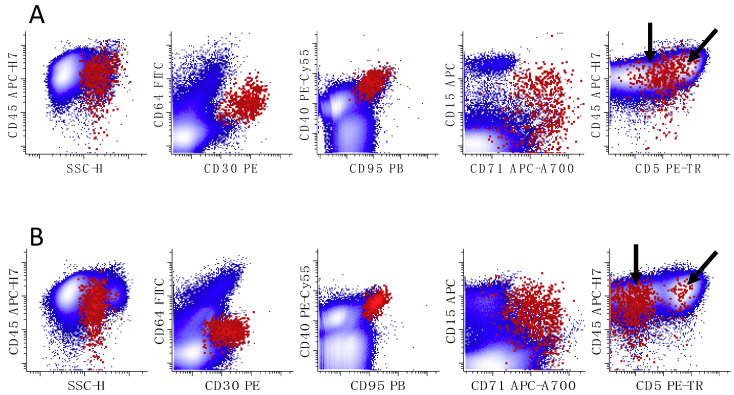
Two representative examples of 9-color FC studies of CHL cases. HRS cells (shown in red and emphasized) are identified by their absence of expression of CD64 (position of negative determined by control experiments, not shown), expression of CD30, CD40, CD95, and increased side light scatter (SSC-H) compared to normal lymphocytes; all remaining viable events are in blue. (**A**) The population of neoplastic HRS cells has expression of CD15 (low to intermediate), CD30 (intermediate), CD40 (intermediate), CD71 (variable), and CD95 (intermediate), without expression of CD20 or CD64. The diagonal relationship between CD45 and CD5 is due to the presence of T-cells bound to the HRS cells (oblique arrow). Unbound “naked” HRS cells are shown with a vertical arrow. (**B**) Neoplastic HRS cells show expression of CD15 (low to intermediate), CD30 (low to intermediate), CD40 (intermediate), CD71 (variable), and CD95 (intermediate), without expression of CD20 or CD64. Distinct rosetted (CD5 positive; oblique arrow) and unrosetted HRS cells (CD5 negative; vertical arrow) are present. All dot plots in A and B show all events. The two cases show similar immunophenotypes, although greater expression of CD5 on the HRS cells of case A suggests a greater degree of T-cell rosetting.

The gating strategy to identify HRS cells of CHL is discussed in other review manuscripts. In summary, CD30, CD40, and CD95 and increased side light scatter are used to identify putative HRS cells; these populations should not show strong CD20 expression [75,78,80]. Due to the presence of T-cell rosetting, gated HRS cells may show a CD3/CD5-positive subset (rosetted fraction) and HRS cells without CD3/CD5 (unrosetted fraction). The unrosetted fraction usually shows low-level CD45 expression (Figure 13).

A prior manuscript from our laboratory evaluated the flow cytometry-derived immunophenotypes of HRS cells in detail [72]. HRS cells uniformly expressed CD30, CD40, CD71, and CD95, while CD15 is expressed in most case (89%). CD40 and CD95 expression on HRS cells was often greater than that on other cellular population in the lymph node. Neoplastic cells, while usually negative for CD19 and CD20, nevertheless can show (usually weak) expression of these two B-cell antigens (in 29% and 35% of cases, respectively). More CD45 expression is identified on HRS cells by flow cytometry compared to immunohistochemistry; on unrosetted HRS cells, some level of CD45 expression was present on HRS cells in 73% of cases. Cases with HRS cells positive for CD45 showed dim expression of the antigen in most cases (64%); however, occasional cases showed HRS cells with CD45 expression at or only slightly below the level observed in the reactive lymphocytes.

We recommend other reviews for a detailed discussion of caveats associated with FC assays for CHL [12,81]. However, one caveat deserves mention here. It is important to note that cells with an immunophenotype compatible with HRS cells may occasionally be identified in tissue biopsies by FC in patients with NHLs, such as some types of T-cell lymphomas [82] and CLL/SLL [83,84,85]. In most cases, a concurrent abnormal non-Hodgkin B- or T-cell population can usually also be identified by FC. In cases were an abnormal B or T-cell population is not identified, CHL could be erroneously diagnosed (although in our experience, this is extremely uncommon). Consequently, the significance of an HRS cell population should be considered in the context of all the FC data. In addition, integration of all the clinical, flow cytometric, morphologic, and immunohistochemical data is critical for proper diagnosis.

Cell populations quantified in lymph nodes involved by CHL as evaluated by FC may have a role in predicting disease prognosis [86]. In a recent study, we identified 62 patients, with B-NHL, T-NHL, and CHL FC studies performed at presentation at our institution with clinical outcome data, after 4–6 cycles of adriamycin, bleomycin, vinblastine and dacarbazine (ABVD) therapy (in all but one patient). Patients were divided into those with a good outcome (sustained complete remission) or bad outcome (disease relapse or primary disease resistance) (mean follow-up, 69.5 months; range, 22–169 months). More HRS-T-cell rosetting at presentation was observed with those who had a sustained complete remission than those with relapse/refractory disease (*p* = 0.022), a finding not influenced by age, histologic subtype of disease, or disease stage. There were trends (but not statistical significance) for increased reactive B-cells (*p* = 0.11), decreased eosinophils (*p* = 0.095), and decreased neutrophils in tissue (*p* = 0.107) being associated with a better prognosis. Both increased B-cells and decreased eosinophils in tissue have been associated with a better prognosis in tissue sections as evaluated by immunohistochemistry [87,88,89]. With regard to the rosetting phenomena, it is generally accepted that CD4^+^ T-cells provide support for the HRS cells to proliferate [90]. It is perhaps possible that patients who present with fewer rosettes have HRS that are more able to proliferate in the absence of T-cell rosettes, possible correlating with the observation of more aggressive disease. Further studies evaluating the influence of the reactive infiltrate on CHL prognosis are ongoing in our laboratory.

## 12. Primary Mediastinal Large B-Cell Lymphoma

Primary mediastinal large B-cell lymphoma (PMLBCL) is a rare type of large B-cell lymphoma that arises in the mediastinum and typically (on initial evaluation) demonstrates the absence of distant tissue involvement. However, the neoplasm at presentation can show infiltration into nearby tissues, such as the lungs and supraclavicular lymph nodes. Neoplastic cells of PMLBCL are typically present in clusters or sheets surrounded by a characteristic fibrous stroma [91,92,93]. PMLBCL and CHL show similar genetic alterations [94,95,96]. Neoplastic cells of PMLBCL are germinal center B-cells demonstrating expression of BCL-6, CD20, and CD79a, without T-cell marker expression. Despite the germinal center origin, CD10 is typically not expressed by the neoplastic cells by immunohistochemistry. Other antigens that are expressed by the neoplastic cells of PMLBCL include CD23, CD30, and MAL [92,93,97].

**Figure 14:**
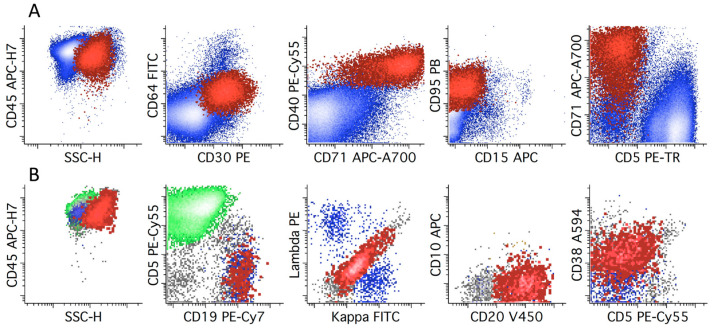
Example of PMLBCL was evaluated by the CHL (**A**) and B-NHL (**B**) flow cytometry assays. (**A**) Neoplastic cells (red; all other events in blue) show expression of CD30 (intermediate), CD40 (intermediate to bright), CD71 (bright), and CD95 (low to intermediate) without CD5, CD15, or CD64 (position of negative determined by prior fluorescence minus one (FMO) experiments) [75]. (**B**) Neoplastic cells of PMLBCL (red) show increased side light scatter (relative to small reactive B-cells [blue]) with expression of CD19, CD20, CD38, and CD45, without CD5, CD10, or surface light chains. Reactive CD5-positive T-cells are in green. Other non-gated events in grey.

The FC combinations for CHL (Table 2) are useful for immunophenotyping PMLBCL. The CHL FC combination allowed for the detection of a neoplastic PMLBCL population in 100% of cases (24 cases examined). Our standard FC combination for B-cell NHL detected the neoplastic population in 75% (18 of 24 cases), suggesting a role for immunophenotyping potential PMLBCL cases with a combination for CHL [98] (example, Figure 14).

Neoplastic cells of PMLBCL showed an immunophenotype by FC similar to that described in paraffin section [92,99,100]. When immunophenotyped by combinations for B-NHL and CHL, neoplastic cells of PMLBCL showed expression of B-cell or B-cell associated antigens (CD19, CD20, and CD40) often without surface light chains. Expression of CD10 was typically not identified (similar to immunohistochemistry [100]). Immunophenotypic similarities to CHL were observed. CD30 was expressed in most cases evaluated (70%)-similar to that reported elsewhere in tissue sections [92]; by FC, CD30 expression on the neoplastic cells of PMLBCL was variable and weaker, compared to the expression of the antigen on HRS cells of CHL [72,75,77]. Like with CHL, neoplastic cells of PMLBCL showed variable expression of CD71 (the transferrin receptor) in all cases in our study. CD15 was not expressed on the neoplastic cells of PMLBCL by FC from any case that was evaluated. Studies from other laboratories by immunohistochemistry showed occasional staining in a paranuclear dot-like for CD15 [92,100]; of note, routine surface staining for FC studies would not detect this intracellular antigen expression.

## 13. Flow Cytometry for Nodular Lymphocyte Predominant Hodgkin Lymphoma

NLPHL is a rare type of B-cell lymphoma, like CHL, that shows scattered, rare, large neoplastic lymphocyte predominant (LP) cells in a nodular, reactive infiltrate that includes small, non-neoplastic lymphocytes and histiocytes [74,101,102,103]. A variety of immunophenotypic and genetic studies confirm that LP cells are a type of germinal center B-cell. The immunophenotype of LP cells by immunohistochemistry show expression of PAX5, CD20, BCL6 (a marker of germinal center origin), and CD45 and lack expression of CD3, CD15, or CD30 [101,103,104]. The typical lack of expression of CD15 and CD30 is in contrast to that seen by HRS cells of CHL [64,74,105,106].

The gating strategy to isolate LP cells by FC is described in detail in our prior manuscript [107]. Two FC tubes are employed to immunophenotype LP cells; CD75 is employed as our prior work has demonstrated uniformity of expression of the antigen on LP cells. CD32 (negative or weakly positive on normal germinal center B-cells) is not expressed or weakly expressed on LP cells, in contrast to moderate expression seen on HRS cells of CHL (see Tube 1; Table 3). T-cell-LP cell rosette formation can be evaluated with CD5 and CD45 in manner similar to CHL. The second tube for NLPHL (Table 3) assesses BCL6 and DNA content (using DAPI) in the cell nucleus. CD10, CD20, CD38, CD40, CD54, CD64 and CD71 are included in both combinations to gate LP cells of NLPHL and to exclude other cellular populations from the analysis.

The two tube assay combination is both sensitive and specific for the LP cells of NLPHL. Seven cases of NLPHL were evaluated with the 2-tube assay combinations and an LP cell population was identified in all 7 cases. Additionally, the 2-tube assay was tested on 9 CHL, 30 reactive and 55 B-NHL cases; 3 cases of B-NHL (DLBCL, NOS) from this series showed a population compatible with NLPHL, while the other 91 cases did not (96.8% specificity) [107].

By FC, LP cells showed a lack of surface light chains. The B-cell markers CD19 and CD20 were expressed on LP cells at the level of normal reactive B-cells; BCL6 and bright expression of CD40 (like CHL) was also observed. LP cells did not show expression CD5, CD10, CD15, or CD64; only minimal CD30 was typically present [103]. The above immunophenotype for LP cells is like that described by other workers by immunohistochemistry.

**Figure 15:**
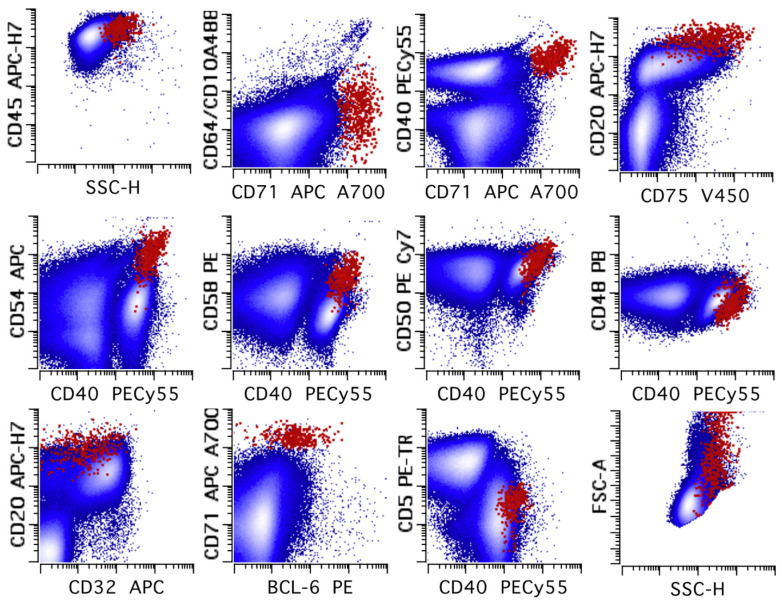
Example of a lymph node involved NLPHL characterized by FC. The large (as determined by increased forward and side light scatter compared to the small lymphocytes) neoplastic LP cells express CD20 (bright), CD40 (intermediate to bright), CD48 (low to absent), CD50 (intermediate to bright), CD54 (intermediate to bright), CD58 (intermediate), CD71 (bright), CD75 (variable), and BCL6 (low to intermediate) without CD5, CD10, or CD64. The immunophenotype identifies a distinct population in multidimensional space. The putative neoplastic LP population is colored in red; all other events are in blue. All events are shown in all dot plots. Reprinted and modified from [107] with permission from Elsevier. See the text and the manuscript on FC for NLPHL [107] for an in depth discussion of the immunophenotype.

This assay for NLPHL allows LP cells to be immunophenotyped by FC. Like with CHL, NLPHL cases can be evaluated for rosettes formation by examining the expression of CD45 and CD5. Fewer T-cell-LP cell rosettes were observed than with CHL (50% of NLPHL cases and 90% of CHL cases showed rosette formation); these prior studies suggest that LP cells interact with T-cells with less avidity than HRS cells. Interestingly, while CD54 was consistently expressed by LP cells, CD58 expression varied from case to case. While we have not rigorously studied the role of CD54 and CD58 in the interaction of T-cells with LP cells, we nevertheless speculate that the inconsistent expression of CD58 is responsible for the less avid interaction of T-cells with LP cells. Our studies identified minimal expression of PD-L1 or PD-L2 on LP cells, suggesting that disrupting of the PD-1/PD-L1/PD-L2 pathway therapeutically likely will be ineffective in NLPHL. Finally, CD32 is either not expressed or weakly on LP cells, similar to normal germinal center B-cells. CD32 is a low-affinity Fc receptor and work by others suggests its expression provides a mechanism of rituximab resistance [108]. Our prior FC studies suggest CD32 mediated resistance will not be operative and consequently rituximab should be useful in treating NLPHL. An example of a case of NLPHL characterized by FC is shown in Figure 15.

## 14. Flow Cytometry for T-Cell/Histiocyte-Rich Large B-Cell Lymphoma

T-cell/histiocyte-rich large B-cell lymphoma (THRLBCL) is an uncommon type of B-NHL, which like CHL and NLPHL, shows a prominent reactive infiltrate and rare neoplastic B-cells. The reactive infiltrate of THRLBCL is primarily comprised of histiocytes and T-cells; reactive B-cells are rare [109,110,111]. Neoplastic cells of NLPHL and THRLBCL show similar germinal center immunophenotypes, demonstrating expression of PAX5, CD20, CD79a, and BCL6, but essentially no expression of CD10 [110,111,112,113].

**Figure 16:**
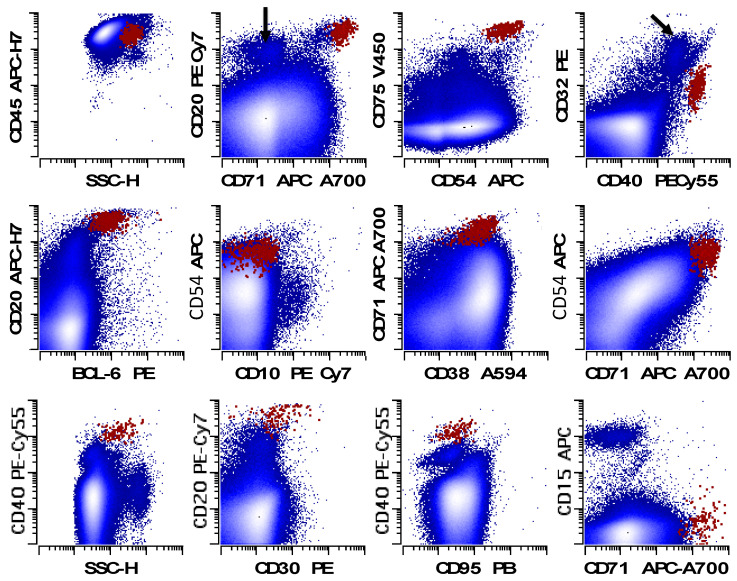
An example of THRLBCL characterized by NLPHL FC panel. The large (as determined by increased forward and side light scatter compared with the small lymphocytes) neoplastic cells express CD20 (bright), CD32 (low to intermediate), CD38 (intermediate), CD40 (bright), CD45 (bright), CD54 (intermediate to bright), CD71 (bright), CD75 (bright), and BCL6 (low to intermediate) without CD5, CD10, or CD64. The neoplastic cells showed no expression of CD15 and little to no expression of CD30 in the CHL FC assay. Normal small B-cells are identified where appropriate with an arrow. Neoplastic cells in red; all other events in blue. (Modified with permission from Glynn and Fromm [114] with permission from Wiley-Liss). Interested readers are directed to this reference for a more detailed discussion of the immunophenotype of THRLBCL by FC.

Flow cytometric studies are challenging to perform due to the rarity of the neoplasm and rarity of the neoplastic cells in involved tissues. In our studies, FC identified the neoplastic cells in 9 of the 11 THRLBCL involved lymph node biopsies (82%) using an assay to immunophenotype CHL and/or a combination of FC tubes designed to immunophenotype NLPHL (Table 3; example Figure 16). NLPHL combinations identified neoplastic cells of THRLBCL in 75% of cases evaluated (six of eight cases) [114]. Criteria to identify these populations is essentially the same as those used for NLPHL (see [77] for details).

Neoplastic cells of THRLBCL by FC showed expression of CD20, CD40, CD75, and BCL6 (germinal center B-cell immunophenotype) but were consistently CD10 negative. Unpublished data from our laboratory suggests that intermediate and high-grade B-cell lymphomas tend to show increased expression of CD71 and likewise, the neoplastic cells of THRLBCL showed increased expression of CD71. In contrast to what is observed with CHL, neoplastic cells of THRLBCL were largely negative for CD15 and CD30. The adhesion macromolecule CD54 was over-expressed, while variable expression of CD58 was noted [77,78,114]. T-cell rosettes in THRLBCL have not been observed by FC. The significance of the expression of CD54 and CD58 in THRLBCL pathogenesis is uncertain. Similarly, the role of CD54 and CD58 mediating interactions of neoplastic cells of THRLBCL with T-cells is unclear. Of interest, however, consistent over-expression of CD54 on neoplastic cells is observed with CHL, NLPHL, and THRLBCL.

## 15. Contribution of Reactive T-Cells to the Diagnosis of CHL, NLPHL and THRLBCL

**Figure 17:**
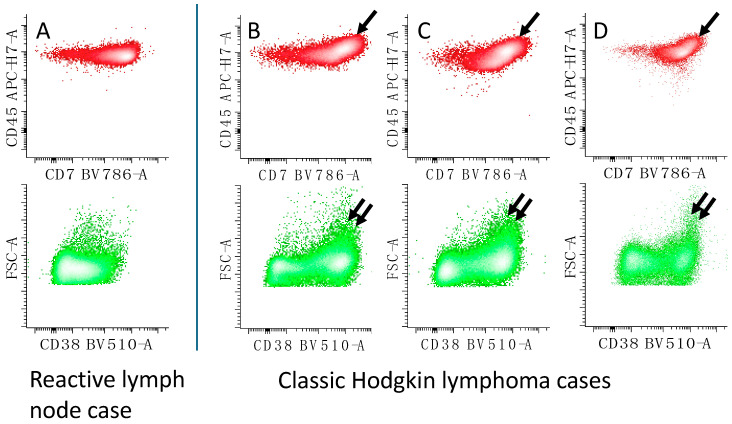
Unique expression profile of reactive CD4^+^ T-cells in CHL. (**B**–**D**) CD4^+^ T-cells from CHL demonstrate increased expression of CD45 and CD7 (single arrows) when compared to a reactive lymph node (**A**). The three examples (**B**–**D**) of CHL cases are from three different patients. This population also shows increased expression of CD38 (suggesting activation) and frequently larger cell size as measured by forward light scatter (double arrows). CD4^+^ T-cells (top panel) are in red; all T-cells (green) from the same case are shown in the bottom panel.

CHL, NLPHL, and THRLBCL are unique when compared to other B-NHL in that the reactive infiltrate predominates, and neoplastic cells are rare. The presence of increased expression of CD45 and CD7 on a CD4^+^ T-cell population (Figure 17) may suggest the diagnosis of CHL [115,116] or THRLBCL [117] in the appropriate clinical and histologic context. Work in our laboratory suggests this population is present in about 76% cases of CHL and 92% cases of THRLBCL; however, it is only present in 8% cases of NLPHL and 4% of reactive cases. The presence of this population with less than approximately 7–10% B-cells, when there is no immunophenotypic evidence of B-NHL (by FC), suggests a diagnosis of THRLBCL over CHL [117] (as T-cells predominate and reactive B-cells are rare in the infiltrate of THRLBCL). The presence of the CD4^+^ T-cell population with increased CD45 and CD7 with more than approximately 10% B-cells favors CHL over THRLBCL.

A recent study from another laboratory identified increased expression of CD71 on CD4-positive T-cells of CHL [118]; this population likely represents the CD4-positive T-cell population with increased expression of CD45 and CD7, described above. Importantly, we and others have shown that NLPHL is characterized by the presence of reactive CD4-positive T-cells which co-express low-level CD8 [117,119,120,121].

## 16. Conclusions

In closing, FC is an indispensable diagnostic technique that can provide diagnostic immunophenotypic information for B-cell NHL, Hodgkin lymphoma, and related neoplasms. Categorization of B-cell NHL relies on expression of CD5 and CD10 for basic classification. However, newer markers are being identified which will undoubtedly improve diagnostic certainty. CHL, NLPHL, and THRLBCL can now be immunophenotyped by FC providing diagnostic information and a powerful research tool. Advances in the field will improve the diagnostic sensitivity and specificity of FC assay for CHL, NLPHL, and THRLBCL, bringing this technique into an ever-expanding number of clinical and research laboratories.

## Figures and Tables

**Table 1:** Immunophenotype of common B-cell neoplasms *.

B-Cell Lymphoma	Immunophenotype
Diffuse large B-cell lymphoma (DLBCL)	Increased forward and side light scatter with variable expression (depending on the case) of CD19, CD20 (variable with some cases showing minimal expression), CD45, CD71 (increased), and monotypic surface light chain. CD10 may or may not be expressed (depending on cell of origin).
Follicular lymphoma (FL)	CD10, CD19 (decreased), CD20, CD38 (decreased), CD44 (decreased), CD45, monotypic surface light chain, increased BCL2 as compared to background T-cells, without CD5 or CD43.
Chronic lymphocytic leukemia/small lymphocytic lymphoma (CLL/SLL)	CD5, CD20 (dim), CD23, CD38 (low), CD43, CD45, mono- or bi-clonal dim to absent surface light chain expression, and CD200 positive, FMC-7 negative; CD81 (dim).
Mantle zone lymphoma (MCL)	CD5, CD20 (normal to bright), CD45, CD81, FMC-7, monotypic surface light chain restriction, without CD23 or CD200.
Burkitt lymphoma (BL)	CD10, CD20, CD38 (increased), CD43, CD45, monotypic surface light chain expression; no over-expression of BCL2.
Hairy cell leukemia (HCL)	Increased forward and side light scatter, CD11c, CD19, CD20 (bright), CD25, CD103, CD123, and monotypic surface light chain expression without CD5 or CD10.
Marginal zone lymphoma (MZL) and lymphoplasmacytic lymphoma (LPL)	CD19, CD20, CD38 (variable), negative for CD5 and CD10. Occasional cases have expression of CD43.

* See reviews elsewhere for details [2,4]. The table lists the most common immunophenotypes; variability between cases exists.

**Table 2:** Combination used to immunophenotype neoplastic cells of CHL and PMLBCL.

Fluorochrome	PB	FITC	PE	PE-TR	PECY5.5	PECY7	APC	APC-A700	APC-H7
	CD95	CD64	CD30	CD5	CD40	CD20	CD15	CD71	CD45

**Table 3:** Combinations used in immunophenotype the neoplastic cells of NLPHL and THRLBCL.

Fluoro-Chrome	V450	FITC/A488	PE	PE-TR	PECY5.5	PECY7	A594	APC	APC-A700	APC-H7
Tube 1	CD75	CD64^+^ CD10	CD32	CD5	CD40	CD20	CD38	CD54	CD71	CD45
Tube 2	DAPI	CD64	BCL-6	CD5	CD40	CD10	CD38	CD54	CD71	CD20

PB, Pacific Blue; FITC, fluorescein isothiocyanate; A488, Alexa488; PE, Phycoerythrin; PE-TR, PE-Texas Red; PE-Cy5.5, PE-Cyanine-5.5; A594, Alexa594; APC, allophycocyanin; APC-A700, APC-AlexaFluor 700. Reprinted and modified from [107] with permission from Elsevier.
